# Advances in human amniotic placenta membrane-derived mesenchymal stromal cells (hAMSCs) for regenerative medicine: enhancing therapeutic potential with biomaterials and scaffolds

**DOI:** 10.3389/fbioe.2025.1590393

**Published:** 2025-09-22

**Authors:** Elvira H. de Laorden, Beatriz L. Rodilla, María Arroyo-Hernández, Maite Iglesias

**Affiliations:** Facultad de C. C. Experimentales, Universidad Francisco de Vitoria, Madrid, Spain

**Keywords:** placenta-stromal-cells, biomaterials, regenerative-medicine, cell-therapy, scaffold, mesenchymal cells

## Abstract

Mesenchymal stromal cells (MSCs) derived from the human placenta amniotic membrane (hAMSCs) have emerged as a promising option in regenerative therapies due to their multipotent differentiation and tissue regeneration capacity, low immunogenicity, and potent immunomodulatory properties. Compared to MSCs from other sources, such as bone marrow or adipose tissue, hAMSCs offer significant advantages, including higher proliferation, lower risk of immune rejection, and greater availability, as their collection is non-invasive and free of ethical concerns. These characteristics make them ideal candidates for regenerative medicine applications and the treatment of degenerative diseases. In this work, we review, from a preclinical perspective, the properties and therapeutic characteristics of hAMSCs derived from the human placenta, and the enhancement in their therapeutic properties when applied in combination with biomaterials such as natural and synthetic polymers or scaffolds, for the treatment of different disorders. The combination of hAMSCs with biomaterials and scaffolds provides a more efficient approach to tissue engineering, enhancing cell viability, proliferation, and integration into damaged tissues. Furthermore, we discuss the properties of scaffolds used to enhance the regenerative capacity of these cells, focusing on their biocompatibility, biodegradability, and ability to mimic the native extracellular matrix. This combined approach has the potential to revolutionize regenerative medicine, providing more effective and personalized therapies for a wide range of chronic and debilitating diseases.

## Introduction

Regenerative medicine aims to repair and regenerate tissues and organs by facilitating the replacement of damaged cells with fully functional counterparts. Mesenchymal Stem Cells (MSCs) have emerged as a keystone in the field of regenerative medicine, offering transformative potential for organ repair and tissue regeneration. These versatile cells have demonstrated significant promise in treating a wide array of diseases and injuries. Among the various sources of MSCs, human Amniotic Mesenchymal Stromal Cells (hAMSCs) derived from the placental membrane have gathered particular attention due to their unique properties and accessibility. Recent progress in regenerative medicine have further underscored the therapeutic potential of hAMSCs, especially when used in conjunction with biomaterials and scaffolds. This synergistic approach has shown enhanced efficacy in promoting tissue repair and regeneration, opening new paths for treating previously challenging medical conditions and injuries.

**FIGURE 1:**
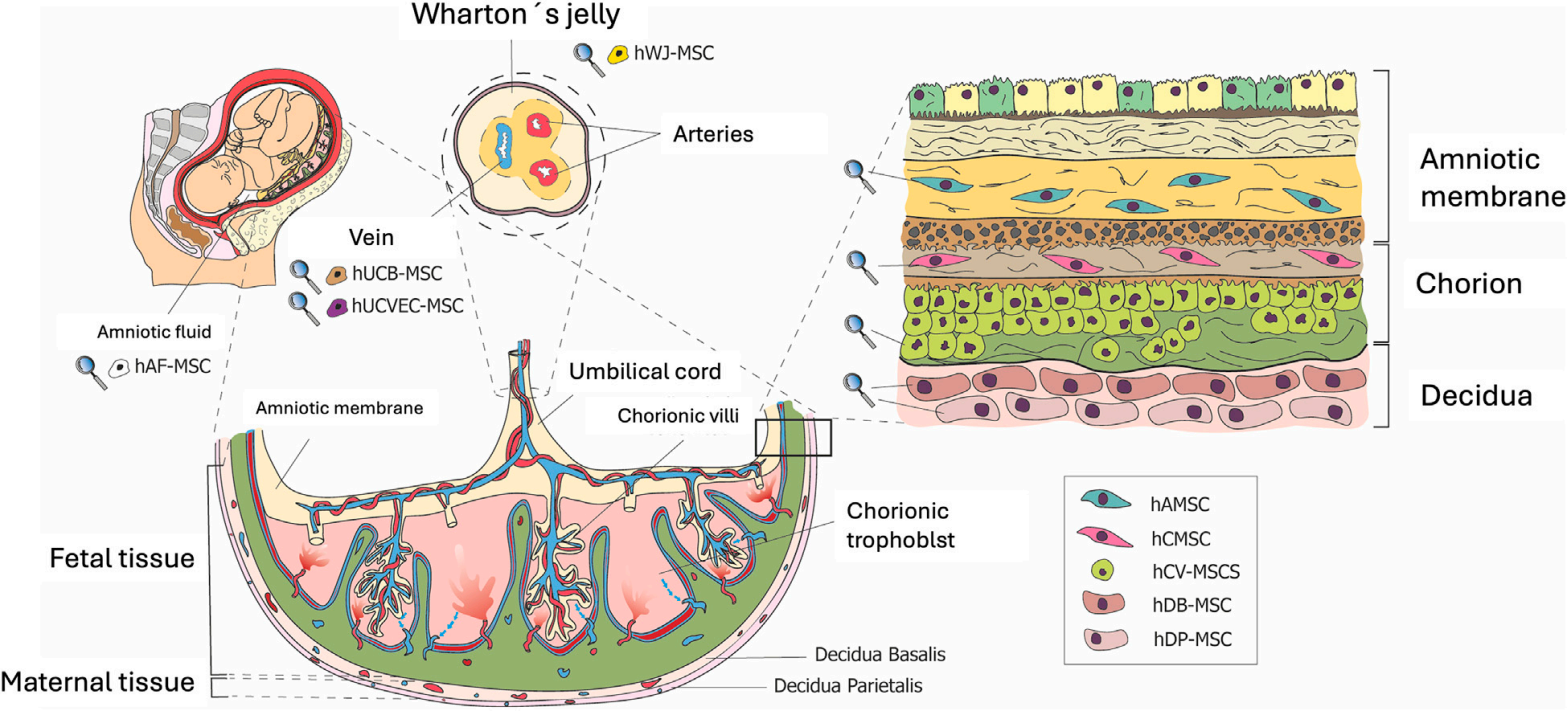
Structure of the full-term human placenta and distribution of perinatal MSCs. Overview of the architecture of fetal annexes and the different MSCs they harbor. The placenta is a disc-shaped structure that surrounds the fetus within the amniotic cavity and contains the amniotic fluid (hAF-MSC, human amniotic fluid mesenchymal stromal cells). The placenta is connected to the fetus through the umbilical cord, which contains two arteries and one vein (hUCVEC-MSC, human umbilical cord vein endothelial cells mesenchymal stromal cells) that transport fetal blood (hUCB-MSC, human umbilical cord blood mesenchymal stromal cells). The arteries and vein are embedded in Wharton’s Jelly (hWJ-MSC, human Wharton’s jelly mesenchymal stromal cells). The umbilical cord is continuous with the amnion (hAMSC, human amniotic mesenchymal stromal cells) and the chorion (hCMSC, human chorionic mesenchymal stromal cells; hCV-MSC, human chorionic villi mesenchymal stromal cells). The maternal component of the placenta is formed by the decidua basalis (hDB-MSC, human decidua basalis mesenchymal stromal cells) and the decidua parietalis (hDP-MSC, human decidua parietalis mesenchymal stromal cells) (Design by Inmaculada Pereda PhD).

The origin of hAMSCs is the human placenta amniotic membrane (Figure 1). The amniotic membrane is a thin, avascular, stratified layer surrounding the fetus, containing amniotic fluid. It protects the fetus from desiccation and pressure and secretes prostaglandins, especially PGE2, during labor (Toda et al., 2007). There are two types of stem cells in the amniotic membrane: i) human amniotic epithelial cells (hAECs), in the innermost layer in contact with the amniotic fluid and derived from the epiblast; and ii) human amniotic mesenchymal stromal cells (hAMSCs), located in the amniotic mesenchyme and derived from the embryonic hypoblast.

hAMSCs, first described in 2004 (In ’t Anker et al., 2004), are isolated from the mesodermal layer of the amnion. They exhibit high isolation efficiency, yielding approximately 5 × 10^8^ hAMSCs per placenta (2 × 10^6^ cells/g of tissue). *In vitro*, hAMSCs are adherent cells with a fibroblast-like morphology, capable of expanding for an average of 14 passages without morphological alterations. Their average cell diameter is 15 µm (ranging from 9 to 24 µm). The hAMSCs display hybrid epithelial-mesenchymal properties at the ultrastructural level, as observed in transmission electron microscopy images (In ’t Anker et al., 2004; Pasquinelli et al., 2007; Parolini et al., 2008; Lindenmair et al., 2012; Pipino et al., 2013). The key characteristics of hAMSCs are summarized in Table 1.

**TABLE 1:** Key properties of hAMSCs.

Property	Description
Origin	Derived from human amniotic membrane (mesodermal layer)
Morphology	Adherent, fibroblast-like; diameter 9–24 µm
Differentiation	Osteogenic, adipogenic, chondrogenic; also neuroglial, hepatic, pancreatic, cardiac, myogenic
Pluripotency Markers	OCT4, SSEA-3, SSEA-4
Tumorigenicity	No evidence of tumor formation; low/no TERT expression
Immunogenicity	Low MHC-I, absent MHC-II; immune-privileged

Comparative studies and literature on hAMSCs face significant challenges due to unclear isolation protocols used in different studies. The lack of precise details on the isolation procedure prevents the exact determination of the placental region from which hAMSCs are extracted. This variability in isolation methods can significantly influence the biological and functional characteristics of isolated cells, complicating the comparative interpretation of data across different studies. In this review, only studies that specify the origin of MSCs in the human amniotic membrane have been analyzed.

The therapeutic potential of hAMSCs is closely linked to their unique properties (Table 2).

**TABLE 2:** Therapeutic applications of hAMSCs.

Application area	Details
Bone/Cartilage Repair	Osteochondral defect repair, cartilage-like tissue regeneration
Fibrosis	Reduces liver, renal, and pulmonary fibrosis; modulates ECM genes
Cardiac/Vascular	Cardiomyocyte differentiation, angiogenesis, improved infarct healing
Skeletal Muscle	VML repair, myogenic differentiation, Wnt signaling
Nervous System	Axonal regeneration, neuroprotection, inflammation reduction
Wound Healing	Promotes fibroblast migration, keratinocyte proliferation, anti-apoptotic effects

## Differentiation potential of hAMSCs

hAMSCs are routinely differentiated *in vitro* into the three mesodermal lineage cell types, indicating osteogenic, adipogenic and chondrogenic differentiation capacity (Portmann-Lanz et al., 2006; Alviano et al., 2007; Kim et al., 2007; Díaz-Prado et al., 2010; Wolbank et al., 2010; Leyva-Leyva et al., 2013). However, hAMSCs express pluripotency markers such as OCT4 (Zhao et al., 2005), SSEA-3, and SSEA-4 (Kim et al., 2011), suggesting they may represent a primitive form of stem cell capable of differentiating into all three germ layers. However, unlike iPSCs, there is no evidence of human tumorigenicity, and telomerase reverse transcriptase (TERT) expression is absent or very low (Mihu et al., 2009).

In addition to this, *in vitro* differentiation of hAMSCs into neuroglial cells (Sakuragawa et al., 2004; Portmann-Lanz et al., 2006; Kim et al., 2007; Chang et al., 2010; Leyva-Leyva et al., 2013), hepatocytes (Tamagawa et al., 2007), pancreatic cells (Tamagawa et al., 2009), nucleus pulposus cells (Ni et al., 2014), cells with phenotype similar to cardiomyocytes *in vitro* and *in vivo* (Zhao et al., 2005; Tsuji et al., 2010), chondrocytes *in vivo* (Wei et al., 2009), smooth muscle cells *in vivo* (Minagawa et al., 2010), and myogenic and angiogenic potential (Portmann-Lanz et al., 2006; Alviano et al., 2007; Kawamichi et al., 2010; Jiang et al., 2015; Zhang Y. et al., 2019) have been described. However, when considering the differentiation capacity of these cells, it is important to consider the impact of the culture conditions in which they are maintained. Manuelpillai group studies demonstrate that serial expansion in xenobiotic-free media alters hAMSCs characteristics, inducing a shift from epithelial to mesenchymal-stromal-like cells, reducing their differentiation capacity, specifically, chondrocyte, hepatocyte, and pancreatic-like cells (Manuelpillai et al., 2011).

## Angiogenic and antifibrotic activity of hAMSCs

**FIGURE 2:**
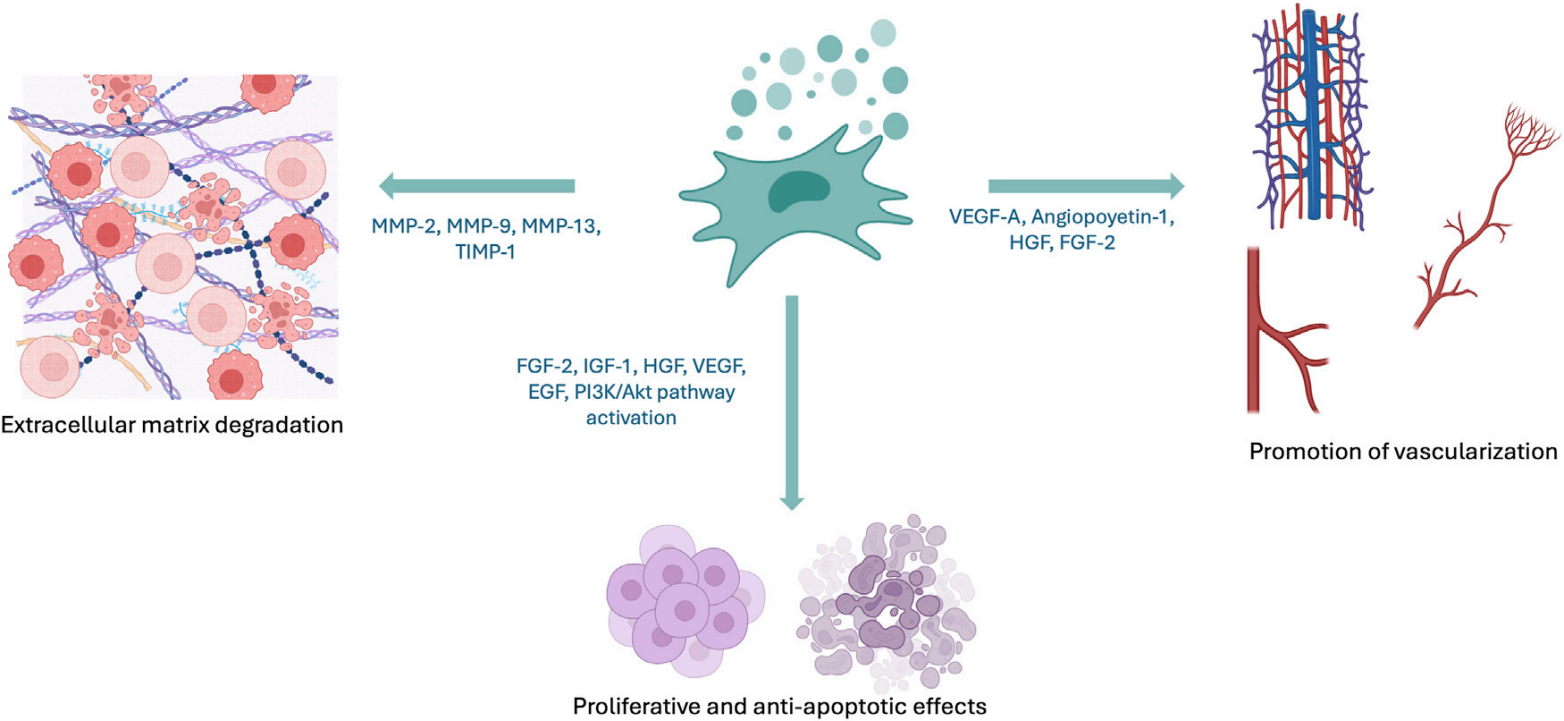
Effects of hAMSCs and their secretome. hAMSCs secrete soluble factors and exosomes that modulate extracellular matrix degradation, vascularization, and apoptosis. Created with BioRender.

hAMSCs exhibit neovascular and angiogenic properties similar to Bone Marrow Mesenchymal Stem Cells (BM-MSC) and enhanced compared to Adipose Tissue-derived Mesenchymal Stem Cells (AT-MSC) (Figure 2). hAMSCs express high levels of pro-angiogenic factors such as VEGF-A, angiopoietin-1, HGF, and FGF-2, and anti-apoptotic factors such as AKT-1 (Alviano et al., 2007; Kim et al., 2011). Some authors relate this capacity to their origin as pericytes. In experiments on endothelial differentiation *in vitro*, hAMSCs undergo morphological and phenotypic changes, but their expression of mature endothelial markers is limited. These studies also show the upregulation of anti-angiogenic genes and downregulation of pro-angiogenic genes in cultures, suggesting a self-protective mechanism against mature endothelial differentiation and that the angiogenic effect exerted by hAMSCs may be indirect (König et al., 2012; Jiang et al., 2015).

hAMSCs can also exert anti-angiogenic effects. Navas et al. evaluated intracameral injection of hAMSCs in a corneal injury model (alkali burn) and observed reduced neovascularization, opacity, inflammatory infiltration, and α-SMA+ cell presence (Navas et al., 2018).

## Proliferative and anti-apoptotic effects of hAMSCs

hAMSCs contribute to wound healing and tissue regeneration through their secretome, by activating pathways such as PI3K/Akt, promoting epidermal cell proliferation, inhibiting apoptosis, and enhancing fibroblast migration (Li et al., 2019) (Figure 2). Additionally, hAMSCs have been utilized for skin organoid generation (Liu et al., 2021). In oncology and reproductive medicine, their secreted cytokines (FGF-2, IGF-1, HGF, VEGF, EGF) have shown proliferative and anti-apoptotic effects in ovarian cancer and premature ovarian failure models (Liu et al., 2021).

## Immunomodulation and immunosuppression of hAMSCs

hAMSCs possess an immune-privileged status, essential for placental function in maternal-fetal tolerance. They exhibit low expression of MHC-I and lack of MHC-II as well as co-stimulatory molecules (CD40, CD80, CD86), thereby evading immune recognition (Insausti et al., 2014). No cases of acute rejection of hAMSCs or hAECs grafts have been reported in either human or animal models (Pipino et al., 2013). hAMSCs have been successfully engrafted into multiple tissues, surviving for at least 2 months in xenotransplanted rat myocardial infarctions (Zhao et al., 2005; Tsuji et al., 2010). Similarly, subfascial and systemic injections in mice and neonatal pigs showed no rejection for up to 61 days (Bailo et al., 2004; Wei et al., 2009).

hAMSCs interact with immune cells, including T and B lymphocytes, NK cells, dendritic cells, monocytes/macrophages, and neutrophils, modulating innate and adaptive responses through cell-cell contact and soluble factors (IL-6, PGE2, TGF-β, NO, IDO, HGF, LIF, IGF-1, galectin-1, adhesion molecules). Their immunosuppressive properties depend on reciprocal interactions with host immune cells. Pro-inflammatory cytokines from T cells and antigen-presenting cells activate hAMSCs, inducing cytokine and chemokine release to regulate immune responses and promote tissue repair. This framework points to a complex, context-dependent immunomodulatory network. Importantly, this bidirectional interaction is crucial; inflammatory cytokines from the host milieu prime hAMSCs to exert anti-inflammatory and reparative actions, positioning them as active participants in immune homeostasis and tissue regeneration (Insausti et al., 2014; Pogozhykh et al., 2018; Bulati et al., 2020; Torre and Flores, 2020; Liu et al., 2021; Nitahara-Kasahara et al., 2023).

hAMSCs inhibit monocyte differentiation into dendritic cells (Insausti et al., 2014) and induces M2 macrophage polarization, suggesting an anti-inflammatory role in various pathologies (Bulati et al., 2020; Nitahara-Kasahara et al., 2023). They also suppress allogeneic T-cell proliferation via direct cell contact and factors like PGE2, IL-10, TGF-β, NO, and IDO. Kang et al. and Wolbank et al. demonstrated that hAMSCs dose-dependently inhibited mitogen-activated peripheral blood mononuclear cell (PBMC) proliferation (Wolbank et al., 2010; Kang et al., 2012). This fact highlights the role of soluble factors and the necessity of cell-cell interaction in hAMSCs-mediated immunomodulation (Wolbank et al., 2010; Kang et al., 2012; Vellasamy et al., 2012; Bulati et al., 2020).

Due to all the aforementioned reasons, hAMSCs constitute a highly attractive source of MSCs for applications in cell therapy and regenerative medicine. While they share the mesenchymal profile of BM-MSCs and AT-MSCs—the most extensively studied MSCs sources—they uniquely express pluripotency markers, suggesting a superior differentiation potential. Importantly, unlike hESCs (human Embryonic Stem Cells) and iPSCs (induced Pluripotent Stem Cells), hAMSCs do not exhibit tumorigenic risk. Their isolation is ethically uncontroversial and does not require invasive or painful procedures for the donor, as the placenta is considered medical waste postpartum. Additionally, hAMSCs are not subject to age-related variability and remain unaffected by environmental stressors. Collectively, these characteristics position hAMSCs as highly promising candidates for the development of advanced MSC-based therapeutic strategies.

Recent studies have demonstrated the efficacy of hAMSCs in promoting tissue regeneration in various preclinical models.

## Bone and cartilage injuries

hAMSCs have been employed in the treatment of bone and cartilage injuries, paving the way for potential clinical applications in both bone and cartilage regeneration. hAMSCs sheets encapsulating cartilage particles have been found to facilitate osteochondral defect repair in rabbits. This approach resulted in the formation of a large amount of hyaline-like cartilage in the defect area, with better integration with surrounding normal cartilage and improved subchondral bone regeneration compared to other treatment groups (Yin et al., 2019; You et al., 2020; Zou et al., 2022).

Results from Muiños-López group show that hAMSCs, when combined with human amniotic membrane (hAM), exhibited better potential for cartilage repair in rabbits, with a reparation capacity similar to that of chondrocytes. Moreover, *in vitro* studies have shown that hAMSCs can be successfully differentiated into chondrocytes using micromass culture systems, suggesting their potential for clinical applications in cartilage repair (Muiños-López et al., 2017).

## Fibrosis

hAMSCs have shown promising potential in the treatment of liver tissue fibrosis: they have demonstrated significant efficacy in reducing liver fibrosis and improving liver function in mouse models by the following facts: i) inhibiting Hepatic Stellate Cell (HSC) activation both *in vivo* and *in vitro*, ii) secreting factors such as insulin-like growth factor binding protein-3 (IGFBP-3), Dickkopf-3 (DKK-3), and Dickkopf-1 (DKK-1), which block the canonical Wnt signaling pathway, and iii) inhibiting collagen deposition and activation of LX-2 cells *in vitro* (Liu et al., 2021). hAMSCs and hAMSCs conditioned medium antifibrotic effects have also been demonstrated in hepatic, renal and pulmonary tissue (Jiang et al., 2015; Liu et al., 2021). It has been described the positive regulation of genes related to extracellular matrix degradation such as MMP-2, MMP-9, MMP-13, and TIMP-1, which could partially explain the protective (and beneficial) effect of hAMSCs (Jiang et al., 2015; Liu et al., 2021).

Through their secretome, hAMSCs participate in the regeneration of skin lesions and wound healing. Additionally, they promote the proliferation of epidermal cells, inhibit apoptosis, and activate the migration of fibroblasts (Li et al., 2019). An exosome-rich conditioned medium (ERCM) from hAMSCs has been shown to improve wound healing through tissue regeneration. This ERCM contains high concentrations of growth factors and neurotrophic factors, which facilitate keratinocyte proliferation for skin repair, activate fibroblasts for extracellular matrix production and regulate angiogenesis and scar tissue formation (Noh et al., 2023).

## Cardiac pathologies

Some preclinical studies and animal models utilize the differentiation potential of hAMSCs for transplantation to replace damaged tissue and restore lost heart function. For example, there was a reduction in infarct size while an improvement in cardiac function *in vivo* in rat myocardial infarction models in which transplanted cells differentiated into phenotypes similar to cardiomyocytes, (Tsuji et al., 2010; Liu et al., 2021). Moreover, the benefits of conditioned medium from hAMSCs have been observed in infarcted hearts of rats through cardioprotection and angiogenesis, reducing myocardial fibrosis area (Danieli et al., 2015).

## Vascular pathologies

Zhang et al. conducted a study investigating the potential of hAMSCs for skeletal muscle regeneration in volumetric muscle loss (VML) (Zhang D. et al., 2019). They induced myogenic differentiation of hAMSCs using 5-azacytidine (5-Aza), a DNA demethylating agent. Myogenic differentiation was confirmed by the expression of skeletal muscle-specific markers (desmin and MyoD) and the involvement of the Wnt/β-catenin signaling pathway. *In vivo*, 5-Aza-induced hAMSCs were implanted into a rat model of VML in the tibialis anterior muscle. Results showed increased angiogenesis and improved local tissue repair in the treatment group. The authors conclude that hAMSCs represent a promising cell source for skeletal muscle tissue engineering applications in VML.

In mouse models, hAMSCs implanted in bladder walls injured by freezing differentiated *in vivo* into smooth muscle cells and facilitated the faster regeneration of smooth stratified muscle structures in damaged bladders (Minagawa et al., 2010). Generally, results obtained in these models are modest regarding cell replacement, with therapeutic effects mainly attributed to soluble factor secretion (Minagawa et al., 2010).

Direct transplantation of hAMSCs into ischemic hind limbs of mice resulted in increased blood perfusion and capillary density, Suggesting that hAMSCs may promote neovascularization (Kim et al., 2012). In other animal models, increased cutaneous blood flow was detected after cell administration (Kim and Choi, 2011).

## Central nervous system

Several studies have demonstrated the efficacy of hAMSCs in Central Nervous System (CNS) repairing, neuroprotection, neuronal regeneration and promoting functional recovery (Kim et al., 2013; Yu et al., 2015; Cho et al., 2018; Giampà et al., 2019; Kulubya et al., 2021; Liu et al., 2021; Hirota et al., 2023; Zhang et al., 2024). hAMSCs intravenous administration has improved functional recovery in rat models of acute traumatic Spinal Cord Injury (SCI) (Zhou et al., 2020; Tsuji et al., 2024). Rats treated with hAMSCs exhibited significant improvements in sensory response and gait function compared to control groups, demonstrating neuroprotective effects and reduced inflammation in these SCI rat models. Other observed effects after hAMSCs treatment were: ED1 macrophages/microglia number decreased, lower levels of inflammatory cytokines such as TNF-α, IL-6, and IL-1β; apoptosis decreased, lowercaspase-3 positive cells and enhanced angiogenesis and axonal regeneration.

Recent studies have demonstrated the potential of hAMSCs in promoting axonal regeneration. In particular, hAMSCs have shown to stimulate axon growth in damaged rat-retina neurons. These cells can help restore neuronal activity in both normoxia and hypoxia conditions (de Laorden et al., 2023).

Despite the increasing number of therapeutic applications of hAMSCs, their clinical use still remains in the early stages, and several important items need to be addressed. Recent advancements have led to novel approaches in tissue engineering using MSCs for organ repair. MSCs are being used in combination with scaffolds and other biomaterials to engineer functional tissue replacements.

## Biomaterials, scaffolds and hAMSCs

Biomaterials are materials that show good properties when interacting with biological systems. They can be natural or specifically designed to replace, treat or enhance the functions of tissues or organs. Their use has grown significantly in recent decades in regenerative medicine, used in tissue engineering and orthopedic medicine, among other areas. In this context, biomaterials combined with MSCs have been proven as one of the most promising approaches for treating various pathologies. The combination of biomaterials and MSCs not only optimizes the biological environment necessary for cellular regeneration but also enhances therapeutic capabilities, improving the integration of implants and scaffolds with the patient’s tissues (Sundelacruz and Kaplan, 2009).

There are different kinds of biomaterials, based on their origin, physical and chemical properties, biocompatibility, and ability to integrate into tissues. According to their origin, biomaterials can be classified as metallic, polymeric, ceramic, composite (combinations of polymers with ceramic or metallic particles), and biological (Vasile et al., 2020).

In the following sections, we will present a structured review of different materials employed as biomaterials, their integration into scaffold systems and their specific applications in combination with hAMSCs (Table 3). This analysis will address the key aspects that determine their suitability for regenerative strategies, including their physical, chemical, and biological properties, as well as their influence on cell behavior and tissue integration.

**TABLE 3:** Types of biomaterials and scaffolds used with hAMSCs.

Biomaterial type	Examples	Applications
Metallic	Titanium, Au-Ni nanowires	Neural interfaces, implants
Polymeric	Hydrogels, GelMA, PPCNg, PLLA/PEG	3D culture, injectable therapies
Ceramics	Hydroxyapatite, Bio-Oss	Bone regeneration
Natural	Collagen, fibrin, chitosan	Soft tissue repair, wound healing
Hybrid/ECM	Decellularized amnion, ECM from hAMSCs	Wound healing, CNS repair

## Metallic, semiconductor and oxides materials

Biomaterials have different applications according to their origin, based on their specific properties. In that sense, for orthopedic medicine, especially in implants and joint prostheses, metallic biomaterials, such as titanium and its alloys and stainless steel, are widely used due to their high mechanical strength and durability (Vayssade and Nagel, 2009). For other applications such as neural interface implants metallic biomaterials such as Platinum (Pt), platinum-iridium (PtIr) alloys, gold (Au), iridium oxide and stainless steel, highly doped semiconductors such as silicon; and ceramics, as titanium nitride and tantalum/tantalum oxide, are some of the materials mostly used due to their outstanding electrical properties. In this vein, current trends in research are exploring the use of new materials as carbon nanotubes (CNTs), graphene and conductive polymers as PEDOT (poly (3,4-ethylenedioxythiophene)) (Kotov et al., 2009).

Besides the surgical trauma, the implantation of biomaterials faces problems related to the high mechanical mismatch in the biomaterial-tissue interface, as they are much more rigid than the surrounding tissue. For example, while neural tissue presents a Young modulus (modulus of elasticity in tension) below 1 kPa, conventional neural interfaces for neural stimulation have Young modulus in the range of several GPa (Rivnay et al., 2017; Wang et al., 2018). For that reason, these materials do not fit soft tissues, as they are not flexible enough.

The former materials can be improved for regenerative medicine uses, by modifications in the bulk or at the surface scale. At bulk scale, devices or implants that contain or are flexible structures facilitate their implantation in hard-to-reach areas, and adapt better to the tissue, improving their contact and adhesion. In addition, their bending features make them ideal to be implanted in zones of repetitive movement as the spinal cord and peripheral nerves, as well as in contact with organs or directly on the skin (Lago and Cester, 2017; Choi et al., 2018). The use of conductive meshes, ribbons and nanowires embedded in polymeric materials have proven their flexibility and adaptability. Furthermore, bulk rigid materials can achieve soft and flexible features if redesigned at the micro or nanoscale. Domínguez et al. have shown how thin gold sheets of 1–1.5 microns in thickness, obtained by pulsed electrodeposition, can be completely bent and unbent with a curvature radius down to 0.3 mm without plastic deformation, formation of cracks, or surface damage (Domínguez-Bajo et al., 2020).

The second approach, surface modification techniques, allows the addition of new properties through modifications at nano- and micrometric scales. One possibility is biofunctionalization which consists in the chemical modification of surfaces to improve the interaction with biological systems. By the immobilization of functional groups, the materials can turn their biocompatibility changing from bioactive to bioinert or reabsorbable (Arroyo-Hernández et al., 2007). Functionalization techniques such Self assembled monolayers are very efficient but highly surface dependent. Nevertheless, other approaches based on organosilane precursors have shown wide range of applications (Manso-Silván et al., 2007; Rezvanian et al., 2016). For example, it is possible to generate specific surface topographies using multiple strategies, such as ion irradiation, laser treatments or lithography (top-down) (Biswas et al., 2012; Abid et al., 2022), or the direct deposition of nanostructures on the surface as template-assisted electrochemical deposition (bottom-up) (Kumar et al., 2017; Peck et al., 2023). It has been demonstrated that these types of nanostructured surfaces enhance the interaction between the biomaterial, cells, and their biological environment (Chapman et al., 2015; Cho et al., 2024). García et al. demonstrated that low-energy Ar+ ion irradiation (1 keV) on the morphology of polycrystalline Ti discs enhances their biocompatibility compared to untreated ones when seeding hAMSCs (Garcia et al., 2023). In a complementary approach, Wang et al. showed that gradient nanostructures generated on titanium surfaces via surface mechanical grinding treatment significantly improve hAMSC adhesion, proliferation and osteogenic differentiation, supporting the relevance of topographical and mechanical surface modification strategies to modulate cell behavior (Cao et al., 2021).

In this vein, surfaces with vertical nanostructures have emerged as promising candidates for cell interfaces with multiple purposes (Zhang et al., 2022). Among the different possibilities, vertical nanowires and cones outstand in this field. Regarding nanowires, Xie et al. showed good neuronal cell pinning by non-invasive nanowires of 150 nm in diameter and 1 μm in height, proposing the nanowires as anchors for cells improving cell adhesion *in vitro* (Xie et al., 2010). Higher viability was reported in HEK-293 (human embryonic kidney cells) cells cultured on substrates with nanowires compared to flat ones (Ryu et al., 2017). Specifically, Liu et al., reported a new vertical nanowire array integrated system, in which the spatial resolution down to sub-micrometer site-to-site spacing, allows electrophysiological recordings from human induced pluripotent stem cells (hiPSCs)-derived neurons, with sensitivity to subthreshold postsynaptic potentials and with signal amplitudes up to 99 mV (Liu et al., 2017). These types of platforms have shown to be critical for understanding the mechanisms of neurological diseases and for developing drugs to treat them. In addition, substrates with standing nanowires are being studied as platforms to promote neural guidance and modulate neural cell activity (Piret et al., 2015; Amin et al., 2018; Domínguez-Bajo et al., 2021), which is of high interest in regenerative medicine and tissue engineering (Hoffman-Kim et al., 2010). Regarding the use of cones as vertical structures, they are also used as drug delivery systems, either to cells, or to deliver cells as treatments (Sonetha et al., 2022). Lee et al., have developed a flexible polymeric array of microneedles-cone shaped, to locally deliver MSCs in tissue for regenerative therapy, with the purpose of overcoming the problems of cell viability, limited migration capacity in the penetration into the target tissue typically of usual deliver by injection (Lee et al., 2020).

Some studies combine the bio-effects of nanostructured surfaces with physical properties coming from the materials. Labusca et al., 2022, have reported, *in vitro*, significant osteogenic increase while not adipogenic differentiation of AT-MSCs on Ni NW exposed to 4 mT magnetic field compared to non-exposed. Besides magnetic actuation is shown to induce AT-MSCs osteogenesis in the absence of external biochemical cues (Labusca et al., 2022). In this sense, devices combining materials arise as promising candidates in the use of biomaterials. Rodilla et al., have obtained flexible metallic electrodes of nanostructured surface containing vertical Ni-Au core-shell nanowires, in which the physicochemical properties of the Ni-core are combined with the non-toxic Au shells, making the interface biocompatible. No difference in the morphology, viability and neuronal differentiation of rat embryonic cortical cells cultured on the neural interfaces were observed when compared to bare Au NW interfaces (Rodilla et al., 2024).

The integration of metallic, semiconductor or oxide-based nanostructures into biodegradable and biocompatible matrices, whether of natural or synthetic origin, has led to the development of advanced scaffold systems with regenerative potential. These hybrid constructs combine the functional advantages of nanomaterials, such as their distinct physical, chemical, and biological properties, with the mechanical flexibility and low stiffness characteristic of polymeric fibers, hydrogels or gelatin-based supports (Gandhimathi et al., 2019; Samadian et al., 2021).

Hashemzadeh et al. used gold nanowires-hydrogels with Young’s modulus of hundreds of kilopascals, to create a tunable biointerface ready to integrate into any organ-on-a-chip and cell chip system. In the presented work, a promoted differentiation of hAMSCs into osteo and chondrogenic lineages is shown by a significant increase in Collagen I and II production. Additionally, there was enhanced calcium mineralization activity and proteoglycans formation after a cultivation period of 2 weeks within the microfluidic device (Hashemzadeh et al., 2020).

## Polymeric materials

Although the use of polymers has therefore been introduced, a thorough review of the polymeric properties in their interaction with hAMSCs is presented in this section.

Polymeric biomaterials are characterized by their versatility and ease of processing (Vasile et al., 2020). Ideally, polymeric scaffolds for tissue engineering applications should have the following characteristics: (i) adequate surface properties to promote cell adhesion, proliferation, and differentiation; (ii) biocompatibility; (iii) high porosity and surface-area-to-volume ratio with an interconnected pore network (for enhance the cell growing and the efficient transport of nutrients and metabolic waste; and (iv) mechanical properties according to *in vivo* stresses (Hosseinkhani et al., 2014).

These materials, such as polyethylene or biodegradable polymers like poly (L-lactic acid) (PLLA), have applications in medical devices, implant coatings, and even tissue engineering, where they are used as scaffolds that enable MSC proliferation and differentiation (Vayssade and Nagel, 2009; Vasile et al., 2020; Chen and Tao, 2022).

The most used polymers in combination with hAMSCs are hydrogels, polymeric biomaterials capable of absorbing large amounts of water without dissolving. Their cross-linked three-dimensional structure allows them to maintain their shape while providing an ideal moist environment for cell growth (Vasile et al., 2020; Guan et al., 2022).

One of the main challenges in the clinical translation of stem cell-based therapies is the need to proliferate a large number of cells for transplant applications, requiring quantities of tens to hundreds of millions per patient. To ensure therapeutic efficacy and according to regulatory standards, these cells must be cultured under strictly defined conditions, minimizing batch-to-batch variability. However, traditional two-dimensional (2D) cell cultures do not accurately replicate the three-dimensional (3D) environment in which stem cells develop within the body (Hosseinkhani et al., 2014; Ravi et al., 2015).

In this context, hydrogels have emerged as a promising platform, as they provide a three-dimensional microenvironment that more closely mimics natural physiological conditions, demonstrating high biocompatibility and low cytotoxicity with hAMSCs. In the last years, biocompatible hydrogels combined with hAMSCs have been designed. They are non-toxic or low-toxic materials, allowing hAMSCs to proliferate actively while maintaining their phenotypic characteristics, stem marker expression, and differentiation capabilities into osteogenic, chondrogenic, and neural lineages (Lv et al., 2014; Li et al., 2020; Saghati et al., 2021; Zheng et al., 2021; Huang et al., 2022).

Additionally, these hydrogels promote a significant increase in the synthesis and secretion of factors such as EGF, bFGF, VEGF, and TGF-β (Wei et al., 2024).

Moreover, the low efficiency of stem cell transplantation and engraftment in recipient tissues is still a key challenge for therapeutic success. There is evidence that many MSCs become trapped in the lungs after systemic intravenous infusion administration. This fact may reduce their therapeutic effect and lead to side effects such as pulmonary embolism (Hoang et al., 2022). Patients with pre-existing kidney problems have developed thromboembolism after the administration of hAMSCs (Moonshi et al., 2022). Additionally, many cells die due to mechanical damage during injection or fail to integrate into the hostile microenvironment of the damaged tissue. During injection, the abrupt increase in fluid velocity from the syringe to the needle exposes the cells to shear forces that compromise their membrane integrity. In this context, injectable hydrogels have emerged as a promising solution by encapsulating stem cells together with bioactive molecules, protecting them from mechanical damage. These hydrogels trap cells and transport them to the injury site, facilitating *in situ* tissue cell colonization, and are highly permeable to oxygen, nutrients, and proteins. Due to their porous and hydrated properties, these biomaterials allow most of the hydrogel to pass through the needle as a solid. Moreover, once injected, hydrogels promote cell retention in the targeted area, preventing the rapid clearance of cells following injection in saline solution, thereby improving viability and graft success in damaged tissue (Madl et al., 2018; Facklam et al., 2020).

An injectable hydrogel composed of GelMA (gelatin methacrylate) has been functionalized with imidazole groups to load hAMSCs and promote their differentiation. Additionally, this gel contains polydopamine (PDA) to carry SDF-1α, which stimulates neuroblast proliferation. The injection of GelMA-imid/SDF-1α/hAMSCs hydrogels in rat models of TBI increases the number of mature neurons to repair TBI damage, with a notable increase (higher expression) in the levels of NeuN, NSE, BDNF, and MAP2 compared to control groups (Zheng et al., 2021).

Huang and collaborators use Poly (polyethylene glycol citrate-co-N-isopropylacrylamide) (PPCN) mixed with gelatin (PPCNg), a thermoresponsive biomaterial that undergoes a reversible phase transition from liquid to solid at 37 °C. The intrauterine transplantation of hAMSCs combined with PPCNg in rat intrauterine endometrial injury models significantly increases cell colonization and utilization rate, promotes structural and functional endometrial recovery, and effectively restores reproductive function. These results suggest that PPCNg can encapsulate cells during transplantation, providing a suitable environment for cell growth, improving survival, and enhancing the utilization rate (Huang et al., 2022).

Cell adhesion peptides have been incorporated into hydrogels that mimic the properties of the native extracellular matrix. As result of this modification there has been an enhance in hAMSC survival, regulation of their fate, and an increase in their paracrine activity, thereby improving their therapeutic efficacy (Zhang et al., 2021). Wei’s group uses an RGDmix hydrogel incorporating the RGDSP peptide (derived from fibronectin) as a synthetic extracellular matrix to encapsulate hAMSCs and evaluates its *in vivo* effect in murine wound healing models. The hydrogel, probably due to moisture retention, increases hAMSC survival at the transplantation site, suggesting that it may enhance the sustained secretion of growth factors, promoting tissue regeneration (Wei et al., 2024).

Biodegradable polymers have the advantage of dissolving in the body in a controlled manner, eliminating the need for surgical removal. Materials that respond to stimuli such as pH or temperature have been developed, tunning their properties, composition, and structure within a defined range (Tong et al., 2018; Vasile et al., 2020). Among these, thermosensitive materials are commonly used for cell culture and release, as their thermosensitivity can be achieved easily and precisely both *in vitro* and *in vivo*. Tong and collaborators used poly (N-isopropylacrylamide) (PNIPAM), a widely studied thermosensitive material, to functionalize the gel, incorporating POSS (polyhedral oligomeric silsesquioxane). In this hybrid gel, hAMSCs adhere and proliferate and can be easily released at low temperatures without damage (Tong et al., 2018).

Lv’s group used a biodegradable poly (L-lactide) (PLLA) scaffold, which exhibits good mechanical properties and excellent biocompatibility. To achieve suitable hydrophobicity for cell adhesion and proliferation, they combined PLLA with poly (ethylene glycol) (PEG). After injecting these hydrogels combined with hAMSCs, they observed improved urethral defect repair in rabbit animal models (including morphology, tissue reconstruction, and complication incidence) and enhanced re-epithelialization. Four weeks after implantation, half of the PLLA/PEG scaffolds were degraded (Lv et al., 2016).

Wang and collegues successfully fabricated a small-diameter vascular graft (SDVG) using PLCL electrospinning loaded with hAMSCs, achieving uniform morphology and high mechanical strength. The grafts demonstrated excellent cyto-, hemo-, and histocompatibility, with enhanced patency and regenerative performance in a rat abdominal aorta model, significantly outperforming cell-free controls. hAMSCs contributed to reduced thrombosis, improved endothelialization, macrophage polarization toward M2, smooth muscle cell recruitment, and extracellular matrix remodeling. Despite promising short-term results, further studies in large animals and over longer implantation periods are required to confirm translational potential. The work highlights the multi-target regenerative mechanism of hAMSCs and supports the feasibility of hAMSC-loaded SDVGs for clinical applications (Wang et al., 2025).

## Ceramic materials and other biomaterials

In the field of bone regeneration, ceramic biomaterials, such as oxide-based functional ceramic materials (Shariati et al., 2022), hydroxyapatite and bioactive glass (Vayssade and Nagel, 2009; Ebrahimi et al., 2022), as well as mineral matrices like Bio-Oss, are used for their ability to promote osteointegration: integration of implants with bone. These materials exhibit excellent biocompatibility and can mimic the structure of natural bone, facilitating bone regeneration. However, Ying and collaborators enhanced the properties of Bio-Oss by combining it with hAMSCs, significantly increasing new bone formation and bone-to-implant contact. They attribute the increased osteogenesis-inducing activity of the transplant to the paracrine activity of hAMSCs (Yin et al., 2019).

There are also natural biomaterials, such as collagen and fibrin, derived from biological sources, which are particularly useful in soft tissue regeneration. These materials have high biocompatibility, facilitating their integration into the body and promoting the healing and regeneration of tissues such as skin and muscles. Despite their excellent biocompatibility, these materials may have limitations in terms of mechanical strength and difficulty in controlling their structure and properties (Wang et al., 2024).

Despite the above-mentioned restrictions, some approaches have developed natural hydrogels combined with hAMSCs. For example, a thermosensitive hybrid hydrogel made of chitosan and hyaluronic acid provided a supportive environment for ASMC proliferation, replenishing damaged cardiomyocytes while simultaneously triggering neovascularization and reducing fibrosis in rat models of myocardial infarction. The observed improvement was attributed by the authors to three possible mechanisms: transdifferentiation of hAMSCs, the creation of a niche for endogenous cell infiltration and biochemical signal release, and a paracrine effect mediated by EVs secreted by hAMSCs (Khan et al., 2022). Additionally, a hybrid scaffold composed of chitosan and carbonate apatite (ChCA) presents a three-dimensional porous structure that promotes hAMSCs proliferation and differentiation into osteoblasts (Kamadjaja et al., 2018).

Silk fiber is another promising biomaterial for combination with hAMSCs due to its unique mechanical properties, good biocompatibility, and adjustable biodegradation (Vayssade and Nagel, 2009; Vasile et al., 2020). Li and collaborators have developed, for the first time, a 3D scaffold, demonstrating high biocompatibility and potential to promote MSC differentiation into the osteogenic lineage both *in vitro* and *in vivo*, as well as the secretion of factors related to angiogenesis (Li et al., 2020).

One of the most important biological scaffolds is the human Amniotic Membrane (hAM) (Wassmer and Berishvili, 2020; Arki et al., 2023; Guo et al., 2024). The structural composition of the human amniotic membrane includes collagen, fibronectin, laminin, and hyaluronic acid, and re its anti-ulcer, anti-inflammatory, and antimicrobial effects has been demonstrated (Wassmer and Berishvili, 2020; Leal-Marin et al., 2021). Varyani and collaborators present a promising and innovative cell-free therapeutic strategy. They hydrogelized decellularized amniotic membrane and immobilized placenta-derived MSC exosomes onto it using sulfo-MBS. Their study confirmed that the hydrogel releases exosomes up to 21 days, leading to an increase in fibroblasts, blood vessels, collagen density, and the expression of genes related to angiogenesis and proliferation in diabetic rat wounds (Varyani et al., 2024). Additionally, scaffolds based on extracellular matrix (ECM) derived from hAMSCs have been developed for restoring damaged tissues of the CNS and to wound healing (Sanluis-Verdes et al., 2017; Protzman et al., 2023; Guo et al., 2024) or combined with other materials for the treatment of bone and cartilage tissue injuries. PLGA (polylactic-co-glycolic acid)-coated ECM derived from hAMSCs was implanted in rat osteochondral lesions, where the transplantation of this scaffold combined with BM-MSCs induced gradual tissue regeneration and promoted cartilage repair (Nogami et al., 2016). Similarly, hAMSC-derived ECM combined with silk fiber enhanced hAMSC proliferation and osteogenic differentiation *in vitro*, while significantly promoting angiogenesis *ex vivo* (Rameshbabu et al., 2020).

**FIGURE 3:**
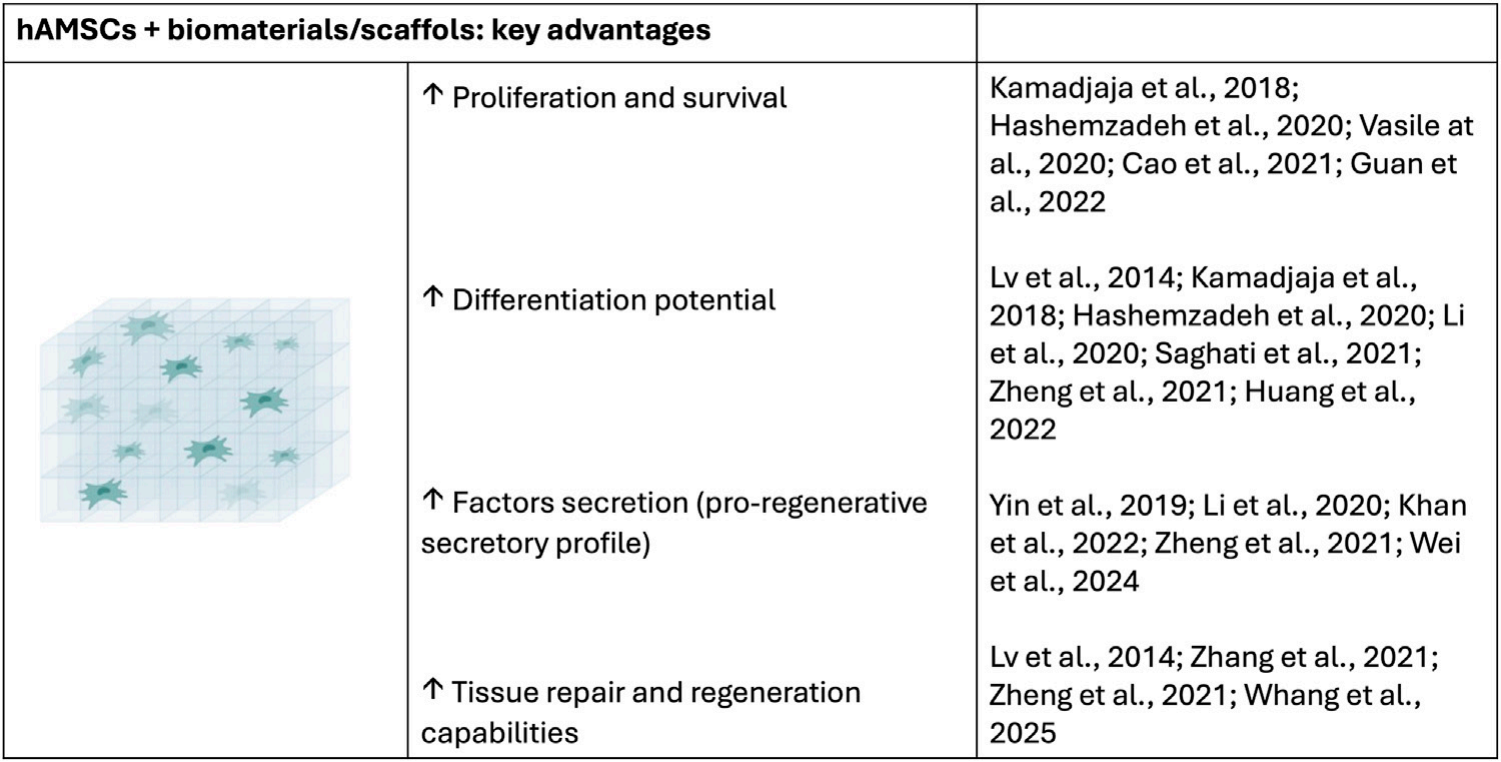
Key advantages of the combination of biomaterials/scaffols with hAMSCs.

In summary, the combination of biomaterials with hAMSCs represents one of the most promising areas of regenerative medicine. In tissue engineering, polymeric and biological biomaterials, when combined with hAMSCs, can create structures that support the regeneration of tissues such as cartilage, skin, and blood vessels. Additionally, biodegradable biomaterials enable the controlled release of growth factors that stimulate hAMSC differentiation, enhancing tissue regeneration and accelerating the healing process. However, the combination of biomaterials with hAMSCs is not without challenges. The integration of these materials into tissues can be complex, especially if the biomaterial degradation rate does not properly align with the natural regeneration process. Furthermore, the physicochemical properties of hydrogels significantly influence cell adhesion, proliferation, and differentiation. Surface modification is essential to enhance biocompatibility and improve interactions with hAMSCs, as their degradability, porosity, and stiffness affect cell behavior. The manufacturing of biomaterials suitable for clinical use can be expensive and technically challenging, limiting their availability. Therefore, the selection of an appropriate hydrogel must consider the impact of its physicochemical properties on cellular responses and the desired therapeutic effect (Figure 3).

In brief, this review provides a comprehensive overview through a preclinical lens, of the current advances in the use of human amniotic membrane-derived mesenchymal stromal cells (hAMSCs) in regenerative medicine, with a particular focus on their differentiation potential, immunomodulatory properties, and therapeutic applications. Special attention is given to the integration of hAMSCs with biomaterials and scaffolds, exploring how these combinations enhance cell viability, tissue repair, and functional recovery across various preclinical models (Table 4). By synthesizing recent findings, this work aims to highlight the translational potential of hAMSC-based therapies and outline key challenges and considerations for their future clinical application.

**TABLE 4:** Current strategies and advances in combining hAMSCs with biomaterials for regenerative medicine applications.

Biomaterial and scaffold	Outcomes/improvements	References
Combination of metallic, semiconductor or oxide nanomaterials in polymers	Higher osteo and chondrogenic differentiation	Hashemzadeh et al. (2020)
Hydrogels	• Retention/preservation hAMSC phenotype, stem marker expression, and differentiation capabilities • Significant/marked increase in the synthesis and secretion of factors	Lv et al. (2014); Li et al. (2020); Saghati et al. (2021); Zheng et al. (2021); Huang et al. (2022); Wei et al. (2024)
Hydrogel composed of GelMA	• Increases the number of mature neurons to repair TBI damage • (higher expression) in the levels of NeuN, NSE, BDNF, and MAP2	Zheng et al. (2021)
RGDmix hydrogel incorporating the RGDSP peptide	Increases hAMSC survival at the transplantation site	Wei et al. (2024)
Biodegradable poly (L-lactide) (PLLA)	Improved urethral defect repair in rabbit animal models (including morphology, tissue reconstruction, and complication incidence) and enhanced re-epithelialization	Lv et al. (2016)
Mineral matrices	Significantly increasing new bone formation (paracrine effect)	Yin et al. (2019)
Chitosan, hyaluronic acid and carbonate apatite hydrogels	• Enhance hAMSC proliferation, promote neovascularization and reduce fibrosis • Higher proliferation and osteo differentiation	Khan et al. (2022); Kamadjaja et al. (2018)
Silk fiber	Promotion osteo differentiation into the in vitro and in vivo, the secretion of angiogenesis related factors	Li et al. (2020)

From a translational perspective, hAMSCs hold promise in treating pathologies such as inflammatory diseases, musculoskeletal conditions, and neurological disorders. However, their successful clinical implementation is closely tied to the development of effective delivery systems that preserve their functionality and viability. In this context, the integration of hAMSCs with biomaterials and scaffolds has gained increasing interest. Biomaterial matrices not only provide mechanical support and structural cues that guide tissue regeneration but also protect the embedded cells from hostile microenvironments. Natural and synthetic scaffolds functionalized with growth factors or extracellular matrix components have been shown to enhance hAMSC proliferation, differentiation, and paracrine activity in preclinical models.

Several *in vivo* studies have demonstrated that combining hAMSCs with advanced biomaterial platforms can significantly improve outcomes in models of skin wound healing, bone defects, and central nervous system injuries by enhancing cell retention, promoting angiogenesis, and accelerating functional recovery. Despite the encouraging evidence from preclinical research, clinical translation remains limited. Key barriers include inter-donor variability, scalability of cell production under Good Manufacturing Practices (GMP) conditions, and the need for standardized protocols to ensure reproducibility and efficacy. Moving forward, the integration of hAMSCs with tissue-specific biomaterials, alongside refinements in cell manufacturing and delivery technologies, will be essential to unlocking their full therapeutic potential and advancing towards clinical implementation.
